# Surfactant/organic solvent free single-step engineering of hybrid graphene-Pt/TiO_2_ nanostructure: Efficient photocatalytic system for the treatment of wastewater coming from textile industries

**DOI:** 10.1038/s41598-018-33108-4

**Published:** 2018-10-02

**Authors:** Zafar Khan Ghouri, Khaled Elsaid, Ahmed Abdala, Saeed Al-Meer, Nasser A. M. Barakat

**Affiliations:** 1 Chemical Engineering Program, Texas A&M University at Qatar, P.O. 23874 Doha, Qatar; 2 Central Laboratories Unit, Qatar University, P. O. Box: 2713 Doha, Qatar; 3 Department of Organic Materials & Fiber Engineering, Chonbuk National University, Jeonju, 54896 Republic of Korea; 4 Department of Chemical Engineering, Minia University, El-Minia, Egypt

**Keywords:** Environmental sciences, Materials science, Nanoscience and technology

## Abstract

In this study, hybrid graphene-Pt/TiO_2_ nanostructure were synthesized by single-step, inexpensive and surfactant/organic solvent free route; hydrothermal technique. The physicochemical properties of hybrid graphene-Pt/TiO_2_ nanostructure were carefully analyzed by multiple techniques, including X-ray diffractometer (XRD), X-ray photoelectron spectroscopy (XPS), field emission scanning electron microscope (FESEM) and transmission electron microscope (TEM). The synthesized hybrid nanostructures were utilized as photocatalyst for the degradation of methylene blue (MB) dye under natural environment at average ambient temperature and mean daily global solar radiation, of about 22–25 °C and 374.9 mWh/cm^2^, respectively. The activity performance indicated considerable degradation of methylene blue (MB) dye and was in the following order Gr (13%), TiO_2_ (60%) and hybrid graphene-Pt/TiO_2_ nanostructure (90%) over 21 min under the natural light illumination. The physiochemical characterization suggests that, the tightly attached metalized TiO_2_ nanoparticles (Pt-TiO_2_) on the high surface area graphene sheets improved utilization of visible light and increased separation and transfer of photo-excited electron (ē) hole (h^+^) pairs. Notably, the hybrid graphene-Pt/TiO_2_ nanostructure exhibited an excellent cyclic stability for methylene blue (MB) dye removal. Finally, the kinetic behavior indicated that the photocatalytic degradation reaction of the dye obeyed the pseudo-first order (Langmuir-Hinshelwood) kinetics model.

## Introduction

The global textile industries are forced to be great, as these are fulfills the clothing requirement of the human being and the synthetic dyestuffs are one of the most common requirements for the textile industry^1,2^. Consequently, the synthetic dyestuffs have become typical industrial organic pollutants during textile dyeing and printing processes^3^. One of these dyes, Methylene blue (MB) is a photoactive basic aniline dye that is not only toxic but also causes serious ecological problems and damage the marine life^4^. Therefore, the removal or degradation of heterocyclic organics pollutants has gradually become hot topics for textile industrialists. Among numerous degradation techniques suitable for dye removal, the heterogeneous photocatalytic process is an efficient and economic technique, which can be successfully used to degrade the organic pollutants and transform them into benign substances^5,6^.

In particular, visible light photocatalysis has appealed great interest because visible light energy takes up more than 80% of the solar energy. Thus the visible light photocatalysis is one of the most promising techniques used to solve environmental remediation^7,8^. However, it remains great challenge to find appropriate photocatalyst that can yield maximum solar light^9^. Among different semiconductors, titanium dioxide have been attracted much attention as a photocatalyst due to its high photosensitivity, nontoxicity and biological and chemical stability^10,11^.

Unfortunately, the photocatalytic activity of TiO_2_ is mainly confined to the UV light due to its wide band gap (3.20 eV). Moreover, easy recombination of photo-induced electron and holes also leads to reduce their quantum yield. Therefore, there is a need to find a solution to activate TiO_2_ under visible light and improve its overall photocatalytic efficiency. To address these technological challenges, defect engineering through metal doping on TiO_2_ and introducing carbonaceous nanomaterials including carbon nanofibers(CNFs), carbon nanotubes (CNTs) and activated carbon (AC) strategy have been applied to fabricate promising photocatalyst to enhance the utilization of visible light for environmental remediation^12–17^.

Graphene, known as two-dimension sp2-hybridized carbon atoms, have a bright future in photocatalysis due to its appealing electronic, thermal and mechanical properties. In addition, graphene has wide surface area (2600 m^2^/g), and high transparency with strong adsorption properties^18–23^. Consequently, the coupling of graphene and TiO_2_ may improve the surface area and light harvesting properties of TiO_2_.

Recently, the incorporation of noble metal nanoparticles into TiO_2_ has gained widespread attention, because it could greatly reduce the recombination rate of photoexcited electrons and holes^24–26^. Therefore, the combination of these three (TiO_2_, graphene and Pt) nanomaterials may become a new type of hybrid nanostructure having high photocatalytic activities. Accordingly, in this study visible light active hybrid graphene-Pt/TiO_2_ nanostructure was synthesized by the hydrothermal technique.

There are already lots of research and development work is done regarding synthesis of graphene-Pt/TiO_2_ nanostructure, but herein, we demonstrate innovative single-step synthesis via surfactant/organic solvent free method.

After carefully studying the physiochemical properties, we investigated the photocatalytic properties of hybrid graphene-Pt/TiO_2_ nanostructure as visible light active photocatalyst by methylene blue degradation.

## Results and Discussion

**Figure 1:**
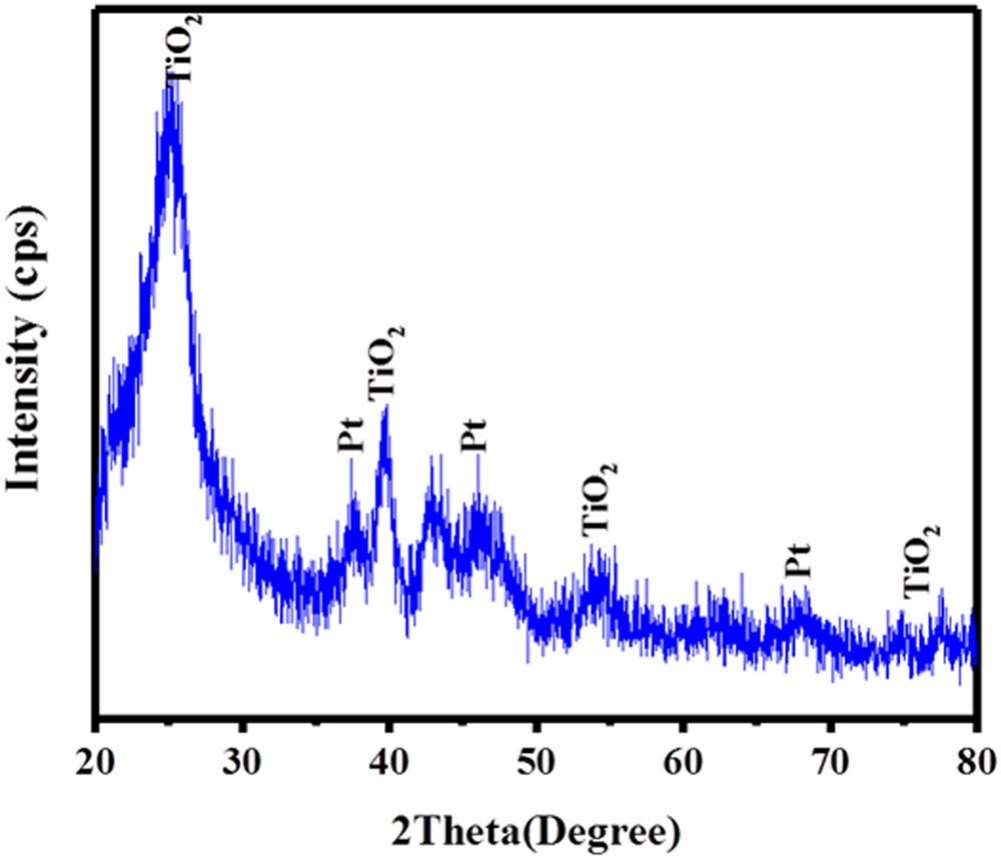
XRD pattern for the synthesized hybrid graphene-Pt/TiO_2_ nanostructure.

The phase and structure of as synthesized hybrid graphene-Pt/TiO_2_ nanostructure was observed by X-ray powder diffraction (XRD). In Fig. 1, the diffraction peaks located at 2θ value could be well indexed to the anatase phase of TiO_2_ (JCPDS no. 21–1272) and cubic crystalline phase of the Pt (JCPDS; #04-0802). There was no detectable peak of graphene, which suggests that, the overlap of characteristic one at 2θ ~ 25° with 101 reflection plane of TiO_2_ at around same 2θ value^27^. Moreover, as expected, the SEM image with its corresponding EDX elemental analysis Fig. 3(A and B) confirms the appearance of carbon (C), oxygen (O), titanium (Ti) and platinum (Pt) atoms.

**Figure 2:**
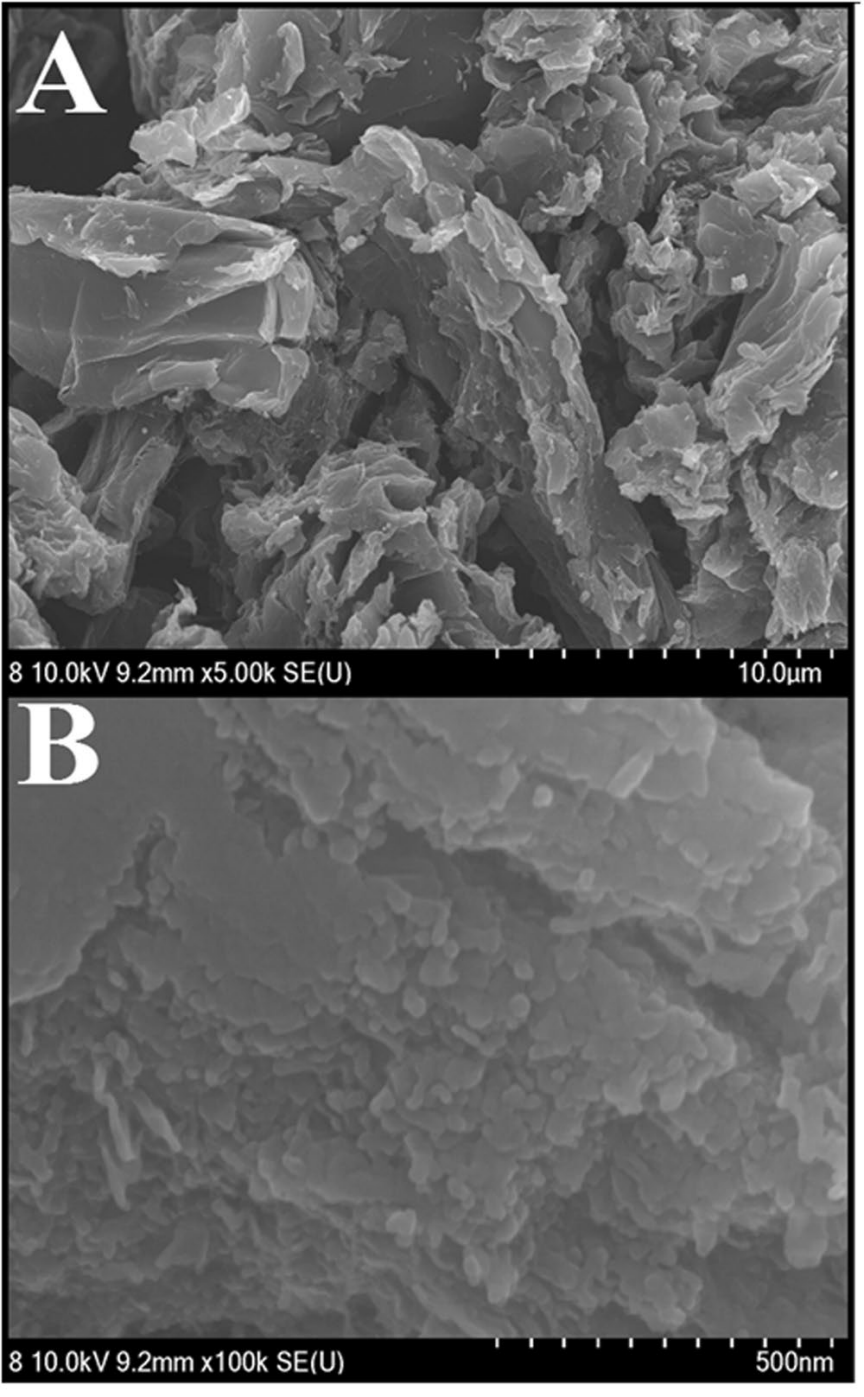
(**A**) Low and (**B**) high magnefication FESEM image for the hybrid graphene-Pt/TiO_2_ nanostructure.

The morphology of the hybrid graphene-Pt/TiO_2_ nanostructure was observed by field emission scanning electron microscopy (FESEM). Figure 2 indicated that natural graphite was successfully converted to graphene. The image (Fig. 2(A)) showed that hybrid nanostructure is mainly consisting of large amount of wrinkled nanosheets which are interconnected to each other’s with slightly curled edges. It can be observed from the high magnification image (Fig. 2(B)) that the Pt/TiO_2_ nanoparticles were heavily dispersed on the surface of graphene.

**Figure 3:**
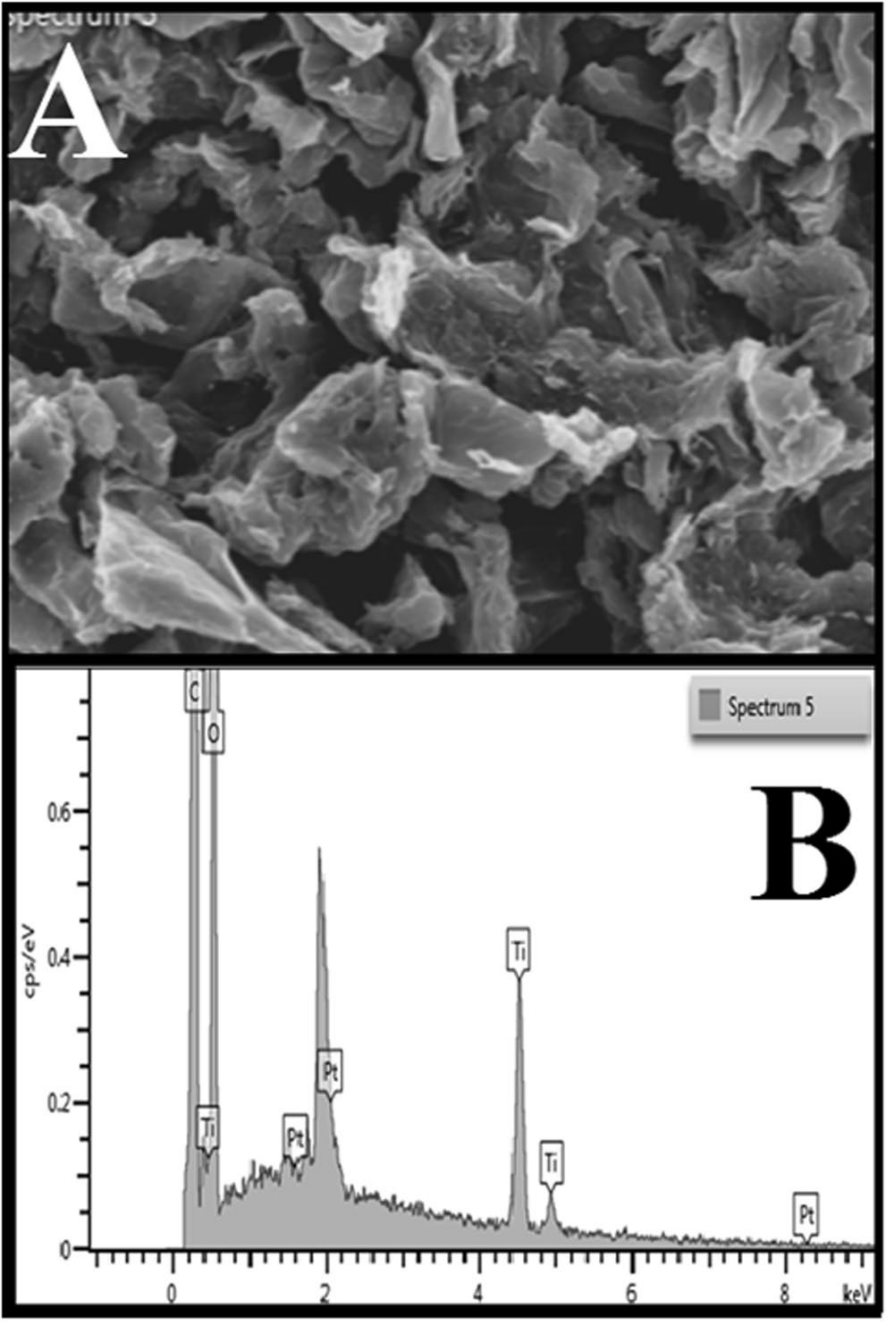
(**A** and **B**) SEM image for the synthesized hybrid graphene-Pt/TiO_2_ nanostructure with corresponding EDS maps.

**Figure 4:**
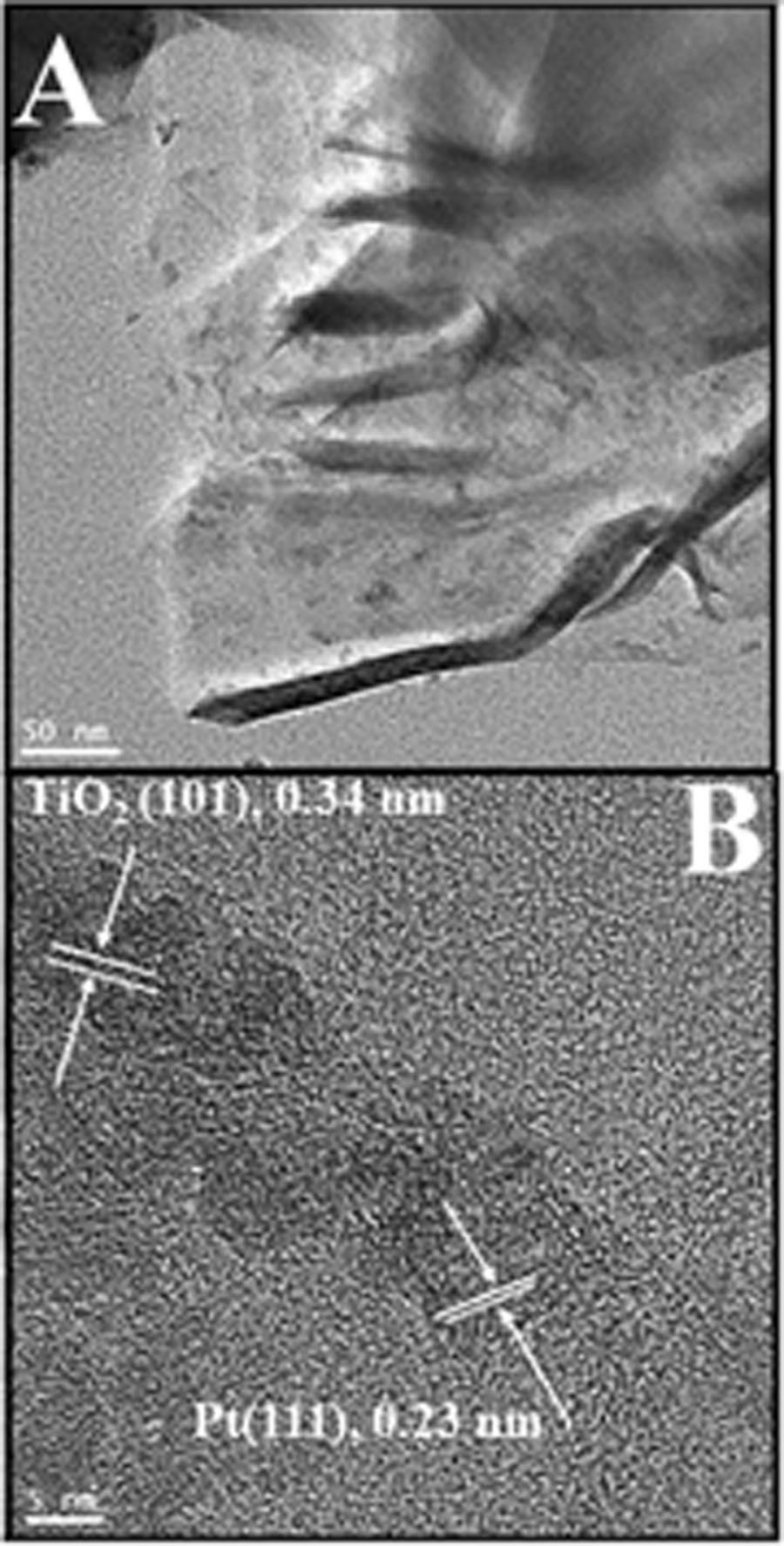
(**A**) TEM, (**B**) HR-TEM image for the synthesized hybrid graphene-Pt/TiO_2_ nanostructure.

Further detailed structure has been observed by transmission electron microcopy (TEM). Figure 4(A and B) showed the low and high magnification images of hybrid graphene-Pt/TiO_2_ nanostructure, respectively. The low magnification image showed that the nanocomposite exhibited the transparent morphology with some folded surface, which suggested the stacking of numerous pieces of graphene, which shows strong π-π interaction on the surface. Moreover, it can be clearly observed from the high magnification image that there are two different kinds of crystalline nanoparticles and their measured planar distance (d = 0.23 nm and 0.34 nm) quite similar with the crystallographic planes of Pt (111) and TiO_2_ (101) confirmed that these nanoparticles are Pt and TiO_2_, respectively.

**Figure 5:**
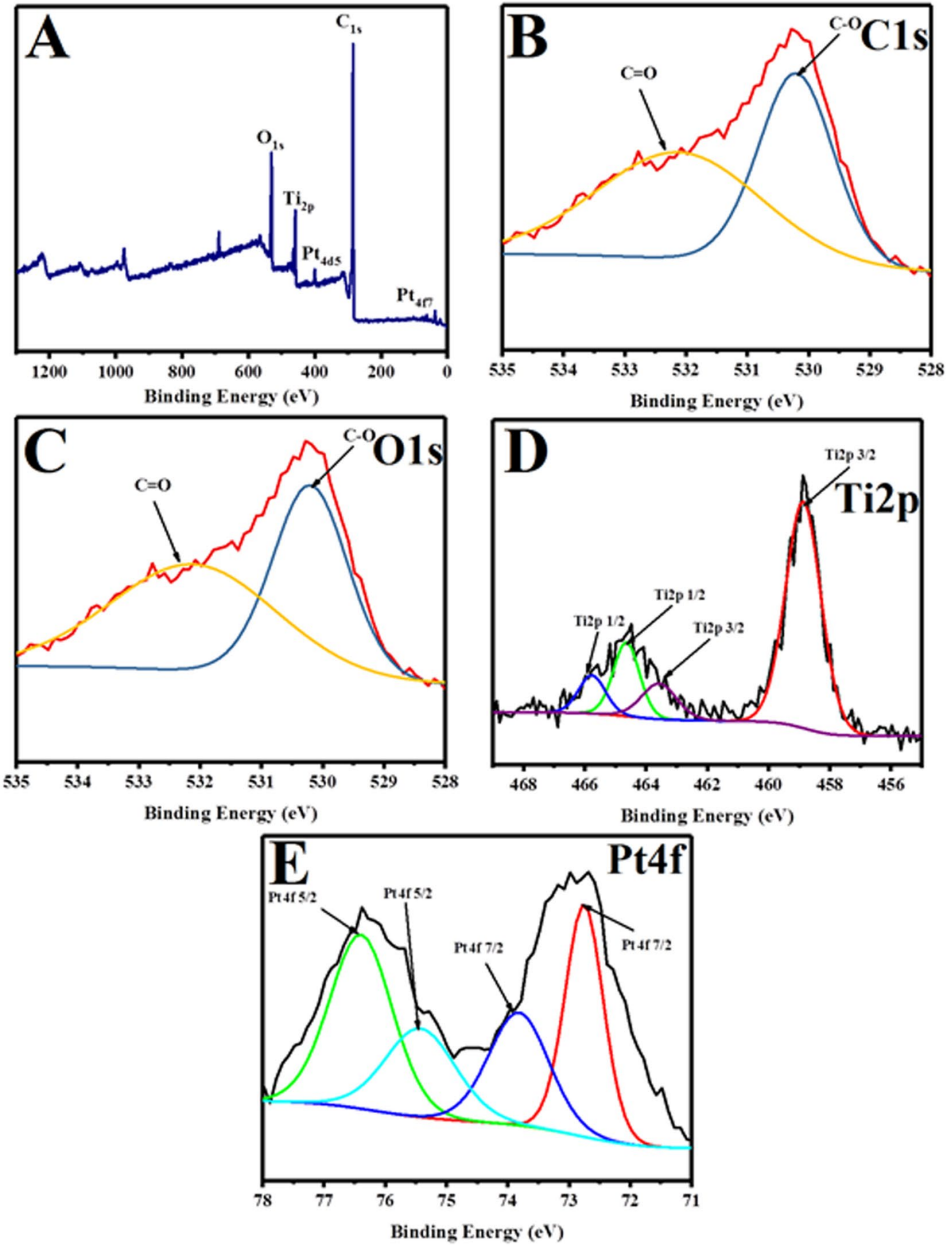
(**A**) XPS spectra survey for the synthesized hybrid graphene-Pt/TiO_2_ nanostructure (**B**) C1s spectra (**C**) O1s spectra (**D**)Ti2p spectra and (**E**) Pt4f spectra for the synthesized hybrid graphene-Pt/TiO_2_ nanostructure.

The surface composition and the valence state of the hybrid graphene-Pt/TiO_2_ nanostructure were investigated using XPS analysis technique. Figure 5(A), displays the corresponding spectrum of synthesized hybrid nanostructure. As expected, in the survey scan the peaks laying at 532.8, 455.6, 314.9, 284.8, and 70.5 eV corresponding to of O_Is_, Ti_2p_, Pt_4d_, C_Is_ and Pt_4f_, respectively confirms the presence of oxygen (O), titanium (Ti), platinum (Pt) and carbon (C) atoms. As shown in Fig. 5(B), deconvolution peaks of the C1s spectrum suggested three peaks. The Peak at 284.0 eV corresponding to C-C bond were generally reflects the sp2 hybridized graphitic carbon atoms^28,29^ while the peak observed at 285.9 eV corresponding to the C-O bond and the peak located at 288.0 eV corresponding to C=O bond were assigned to the oxygen bound species^30^. Plus, the O_1s_ spectrum in Fig. 5(C) confirms the presence of oxygen in metal oxide at the peaks of 530.2 and 533.11 eV corresponding to the Ti-O-Ti and Ti-O-C bonds, respectively in a hybrid graphene-Pt/TiO_2_ nanostructure^31,32^. Deconvolution of Ti_2p_ in hybrid nanostructure (Fig. 5(D)) showed the presence of two set of characteristic peaks, one set centered at 464.5 and 458.9 eV which were assigned to the Ti_2p1/2_ and Ti_2p3/2_ spin-orbital splitting photoelectrons, respectively^31,33^. The observed spin-orbit splitting of Ti_2p1/2_ and Ti_2p3/2_ (5.9 eV) can prove the presence of Ti^4+^ chemical state in the hybrid nanostructure. This indicates that titanium exists in the form of TiO_2_.^34^. While other set of peaks centered at 465.8 and 463.0 eV were attributed to Ti_2p1/2_ and Ti_2p3/2_ spin-orbital splitting photoelectrons, respectively confirm the presence of C-Ti bonds in between TiO_2_ and graphene^28,33^. At last, deconvolution of Pt_4f_ in the hybrid nanostructure (Fig. 5(E)) showed the existence of two pairs of doublets of 7/2 and 5/2^35^. The binding energies of Pt_4f 7/2_ at 73.3 and of Pt_4f5/2_ at 74.8 eV were attributed to metallic Pt. The binding energy of Pt°_4f_ was slightly higher than the reported value^36,37^ which referred to the small particle size and the attachment of Pt° with the TiO_2_/graphene support. The binding energies of Pt_4f5/2_ at 72.6 and of Pt_4f7/2_ at 76.3 eV could be corresponded to Pt^2+^ and Pt^4+^, respectively, thereby indicates that the mixed-valance Pt nanoparticles coexist in the hybrid graphene-Pt/TiO_2_ nanostructure. The formation of mixed-valance Pt nanoparticles indicates the decomposition of H_2_PtCl_6_ in the hybrid nanostructure as follows.

$${{\rm{H}}}_{{\rm{2}}}{{\rm{PtCl}}}_{{\rm{6}}}\to {{\rm{PtO}}}_{{\rm{2}}}\to {\rm{PtO}}\to {\rm{Pt}}$$

In addition, the existence of Pt^2+^ states indicating Pt-O bond which anchored on the surface of TiO_2_/graphene network^38,39^.

**Figure 6:**
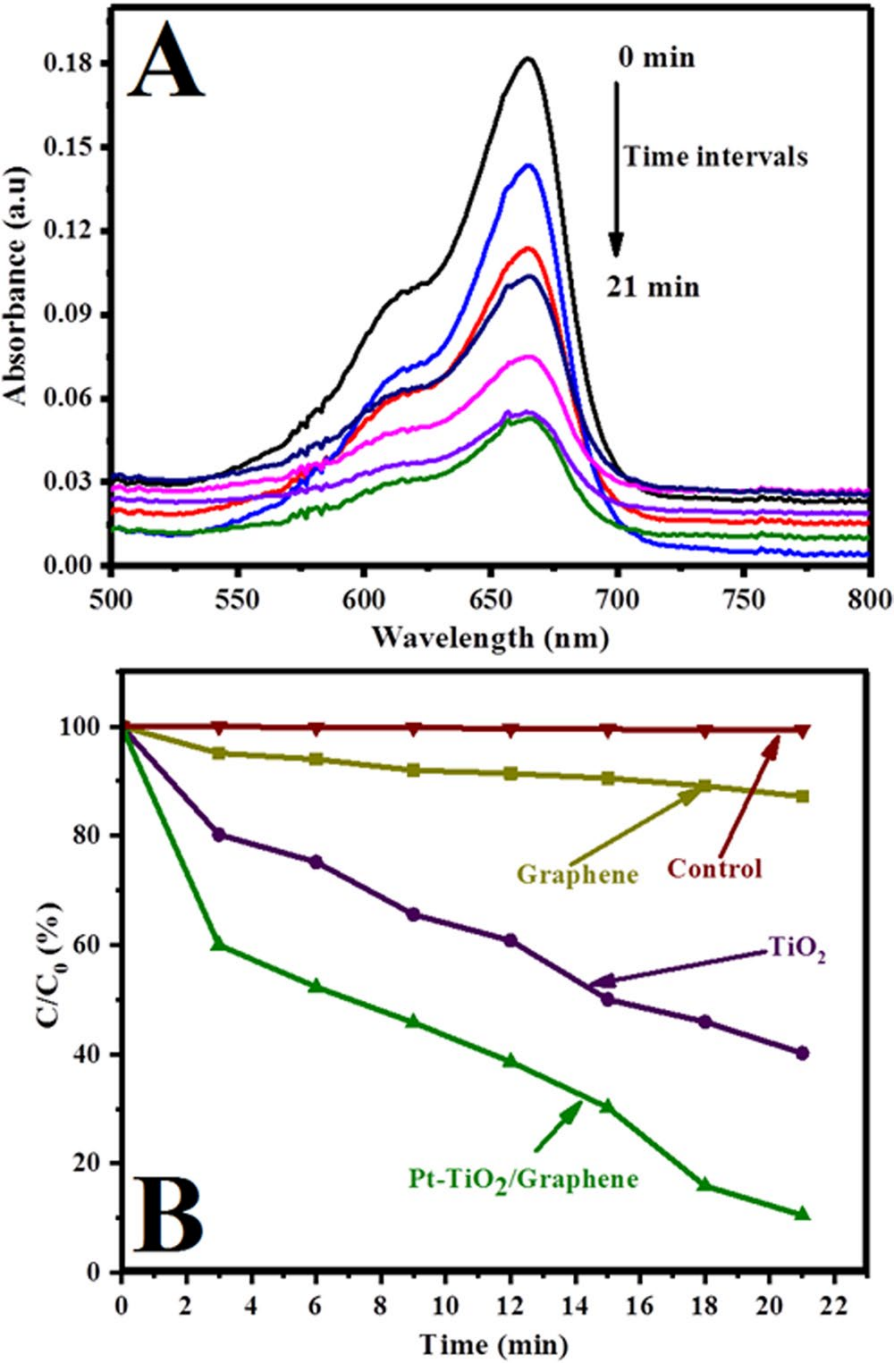
(**A**) Absorption change of methylene blue during the photocatalytic process and (**B**) photocatalytic degradation of methylene blue aqueous solution over the synthesized hybrid graphene-Pt/TiO_2_ nanostructure under visible-light irradiation.

Photocatalytic degradation of methylene blue (MB) using hybrid graphene-Pt/TiO_2_ nanostructure was carried out under natural light illumination, and their comparative studies have been performed with the pristine TiO_2_ and graphene. In addition the experiment on controlled sample without photo-catalyst was also carried out under the identical conditions. Figure 6(A) shows the relationship between absorbance and exposure time of methylene blue (MB) dye solution over hybrid nanostructure. In presence of hybrid graphene-Pt/TiO_2_ nanostructure, the absorption peak intensity of methylene blue (MB) dye solution at λ = 664 nm has noticeably decreased upon increasing the exposure time and it is observed that the most of methylene blue (MB) dye molecules decomposes after 21 min of irradiation time. No further shifting of absorption peak of methylene blue (MB) dye solution can be understood by the decomposition of chromophores^40^. Additionally, Fig. 6(B) shows the comparative study of the degradation of methylene blue (MB) dyes over the blank, TiO_2_, graphene and hybrid graphene-Pt/TiO_2_ nanostructure. The photocatalytic degradation efficiency was calculated using the following formula^4^.

$$MB\,degradad\,( \% )=(\frac{{C}_{0}-{C}_{t}}{{C}_{0}})100$$

where *C*_0_ and *C*_*t*_ are the initial and final concentration based on the absorbance intensity of the sample at a specific time interval. Graphene, TiO_2_ and synthesized hybrid graphene-Pt/TiO_2_ nanostructure exhibits photocatalytic ability for MB dyes solution degradation under natural light illumination in comparison to controlled sample. As shown, the degradation efficiency for pristine TiO_2_ increased slightly compared to those for the pristine graphene sample. However, low photocatalytic efficiency of pristine TiO_2_ is understandable due to its large band gap and partial utilization of visible light energy^41,42^. In contrast, hybrid graphene-Pt/TiO_2_ nanostructure had very high photocatalytic efficiency for MB dyes solution degradation. The decomposition of MB by graphene, TiO_2_ and hybrid graphene-Pt/TiO_2_ nanostructure over 21 min under illumination of natural light were found to be 13%, 60% and 90%, respectively. The observed high photocatalytic efficiency of hybrid graphene-Pt/TiO_2_ nanostructure is understandable because of high surface area which provides more active sites for photocatalytic reactions^43^, additionally graphene could accelerate migration rate of the photoexcited charges therefore helping faster availability to the reaction channels and improving the initiation of the photocatalytic reaction^44^. Moreover, tightly attached metalized TiO_2_ nanoparticles (Pt-TiO_2_) could not only help to activate the hybrid nanostructure under the irradiation of natural light but it can played main function in the separation of photo-excited electron hole pair. In addition, the interfacial region between the metal (Pt) and semiconductors (TiO_2_), which is known as Schottky barrier also attribute to the high photocatalytic activity because it would be beneficial for providing efficient channeling of excited electrons and helps to lower the electron density in the semiconductors (TiO_2_) nanoparticles^45–47^. Subsequently, prevent the electron-hole pair recombination.

In order to determine the kinetic behavior of photocatalyst, the variation of −ln Ct/C_0_ was plotted versus irradiation time, it was turns out that the photodegradation of the dye obeys the pseudo-first order (Langmuir-Hinshelwood) kinetics model:

$$-\frac{{\rm{dc}}}{{\rm{dt}}}={\rm{kC}}$$

where C is the concentration of MB dye solution with respect to time and k (min^−1^) is the observed rate constant, and after doing the integration following relationship will be obtained

$$\mathrm{ln}(\frac{{{\rm{C}}}_{0}}{{\rm{C}}})={\rm{kt}}$$

**Figure 7:**
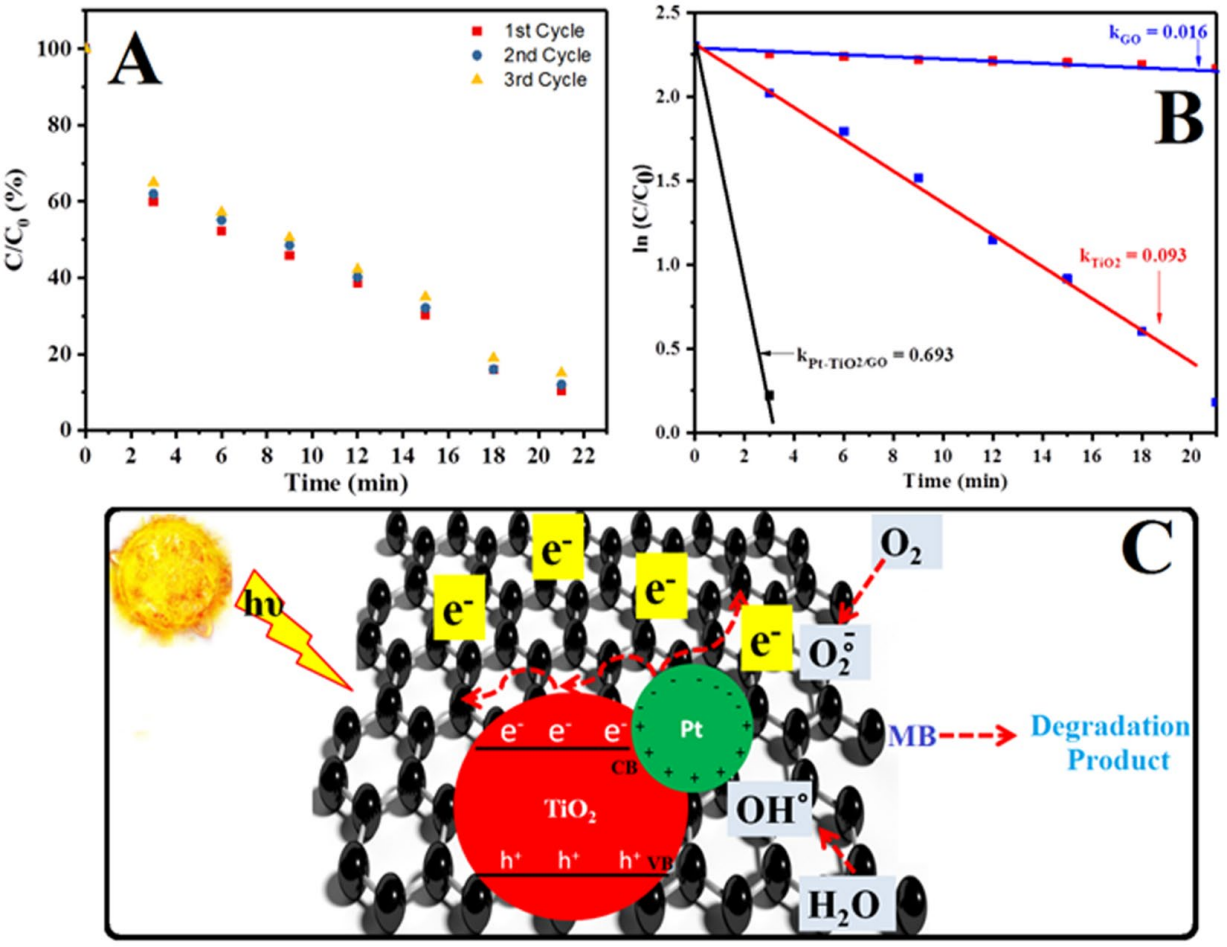
(**A**) Cyclic stability (**B**) rate kinetics for the synthesized hybrid graphene-Pt/TiO_2_ nanostructure under visible-light irradiation and (**C**) schematic illustration of the possible photocatalytic mechanism of synthesized hybrid graphene-Pt/TiO_2_ nanostructure under natural light irradiation.

From Fig. 7(B), it was found that the degradation rate constant obtained for hybrid graphene-Pt/TiO_2_ nanostructure was comparatively very high. Further, the cyclic stability of hybrid graphene-Pt/TiO_2_ nanostructure was evaluated by repeated photodegradation of MB dye solution (three times). The results from Fig. 7(A), demonstrates almost similar photocatalytic behavior of the nanocomposite. The possible mechanism of photocatalytic degradation of methylene blue under irradiation of natural light can be described as follows (Fig. 7(C)). The TiO_2_ nanoparticles absorb visible light, resulting the electrons (e^−^) in the valence band (VB) of TiO_2_ nanoparticles can be excited to conduction band (CB) of TiO_2_ nanoparticles. As a result, electron hole (h^+^) is generated in valance band (VB). Due to good electrical conductivity of graphene the excited electron (e^−^) can moved freely to the graphene through interface^48–51^. Further the electron (e^−^) may be trapped by the Pt nanoparticles and contributed to the improving separation of photoexcited electron-hole pairs^12^. On the other hand, due to the synergistic effect of mixed-valance Pt nanoparticles, the electron (e^−^) from the Pt nanoparticles where also excited from conduction band (CB) and creating hole (h^+^)^25^. Moreover, the recombination rate of charge pairs can be diminished by Pt nanoparticles. Finally, the electron can react with O_2_ and generate super oxide (${O}_{2}^{.-}$) radicals while remaining holes (h^+^) can react with water or OH^−^ ions to generate an extremely oxidizing radical ($O{H}^{.}$) which was mainly responsible for degradation of methylene blue dye.

## Conclusions

Hybrid graphene Pt/TiO_2_ nanostructure can be successfully synthesized using single-step, low-cost and environment friendly hydrothermal technique. The hybrid nanostructure was well characterized by XRD, SEM, TEM and XPS techniques. Single-step without use of organic solvents/surfactant synthesis and effectively utilization of synthesized hybrid graphene Pt/TiO_2_ nanostructure as a natural light sensitive photocatalyst was the main objective of the work. Therefore, the decomposition of model pollutant (methylene blue) using synthesized hybrid nanostructure reaches 90% just over a 19 min of irradiation. It is suggested that the robust synergetic interaction between metalized semiconductor (Pt-TiO_2_) and graphene contribute to the enhancement of photocatalysis. More importantly, as compared bare TiO_2_ and graphene, synthesized hybrid graphene Pt/TiO_2_ nanostructure exhibit high rate constant with good stability over three cycles. Finally, synthesized hybrid graphene Pt/TiO_2_ nanostructure can be a potential photocatalyst for environmental remediation.

## Experimental

### Synthesis of Graphene

Graphene oxide was prepared by the following procedure using the modified Hummers method through oxidation of natural graphite^52^. Firstly, 100 ml of previously cooled concentrated sulphuric acid (H_2_SO_4_) and 5 gm of natural graphite powder was taken in a round bottom flask under vigorous stirring until the homogeneous dispersion is obtained. Secondly, 12 g of potassium permanganate (KMnO_4_) was added slowly into the solution under the stirring for 2 h, followed by addition of 150 ml DI water into the dispersion under the stirring for another 1 h. Then above dispersion was treated with 45 ml of 30wt. % hydrogen peroxide (H_2_O_2_) so as to terminate the reaction. Then, as prepared product was filtered and washed repeatedly with dilute hydrochloric acid (HCl) and deionized water to neutralize the pH. The resulting product was dispersed in water to give a brown dispersion. Finally, the dispersion was filtered and dried in vacuum oven for overnight at 60 °C.

### Synthesis of hybrid graphene-Pt/TiO_2_ nanostructure

**Figure 8:**
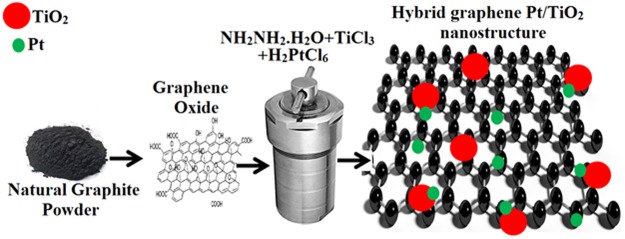
Schematic diagram for the whole synthesis procedure.

Hybrid graphene-Pt/TiO_2_ nanostructure was synthesized by hydrothermal method. Firstly, 1 gm of previously synthesized graphene oxide (GO) was exfoliated in 150 mL deionized water by ultrasonication for 1 to 1.5 h. Secondly, 75 µL of 8% hydrogen hexachloroplatinate (H_2_PtCl_6_) solution and 125 µL titanium chloride (TiCl_3_) solution was added into the above suspension by ultrasonication for 30–45 min in a water bath, followed by addition of 200 µL of 98% hydrazine monohydrate (NH_2_NH_2_.H_2_O) solution into the mixture. Then, the reaction mixture was treated in a Teflon vessel sealed in an autoclave equipped with microwave heating system at 150 °C for 8 h. After naturally cooling the prepared hybrid nanostructure was washed, filtered and dried in vacuum oven for overnight at 60 °C. The final product, hybrid graphene-Pt/TiO_2_ nanostructure was collected and characterized. Figure 8 shows a schematic illustration for the synthesis procedure and the final product.

### Characterization and photocatalytic investigations

The physicochemical properties of the final product were analyzed by powder X-ray diffraction (XRD, Rigaku Japan), field-emission scanning electron microscopy (FESEM, Hitachi S-7400, Japan), transmission electron microscope (TEM, JEOL JEM-2200FS, Japan) and X-ray photoelectron spectroscopy (XPS, AXIS-NOVA, Krotas Analytical, UK) techniques. While photocatalytic activity investigation of synthesized hybrid graphene-Pt/TiO_2_ nanostructure was evaluated under natural environment on sunny day at average ambient temperature and mean daily global solar radiation, of about 22–25 °C and  374.9 mWh/cm^2^, respectively in January between 12:00 p.m. to 02:00 p.m. by degradation of methylene blue (MB) dye solution. The procedure used for photocatalytic investigation of synthesized hybrid graphene-Pt/TiO_2_ nanostructure in this study has followed according to our previous studies^4–6^.
