# Author Correction: Surfactant/organic solvent free single-step engineering of hybrid graphene-Pt/TiO_2_ nanostructure: Efficient photocatalytic system for the treatment of wastewater coming from textile industries

**DOI:** 10.1038/s41598-019-44276-2

**Published:** 2019-05-22

**Authors:** Zafar Khan Ghouri, Khaled Elsaid, Ahmed Abdala, Saeed Al-Meer, Nasser A. M. Barakat

**Affiliations:** 1 Chemical Engineering Program, Texas A&M University at Qatar, P.O. 23874, Doha, Qatar; 2 Central Laboratories Unit, Qatar University, P. O. Box: 2713, Doha, Qatar; 3 Department of Organic Materials & Fiber Engineering, Chonbuk National University, Jeonju, 54896 Republic of Korea; 4 Department of Chemical Engineering, Minia University, El-Minia, Egypt

Correction to: *Scientific Reports* 10.1038/s41598-018-33108-4, published online 02 October 2018

In Figure 5B, the XPS spectra are incorrect. The correct Figure 5 appears below as Fig. 1.

**Figure 1:**
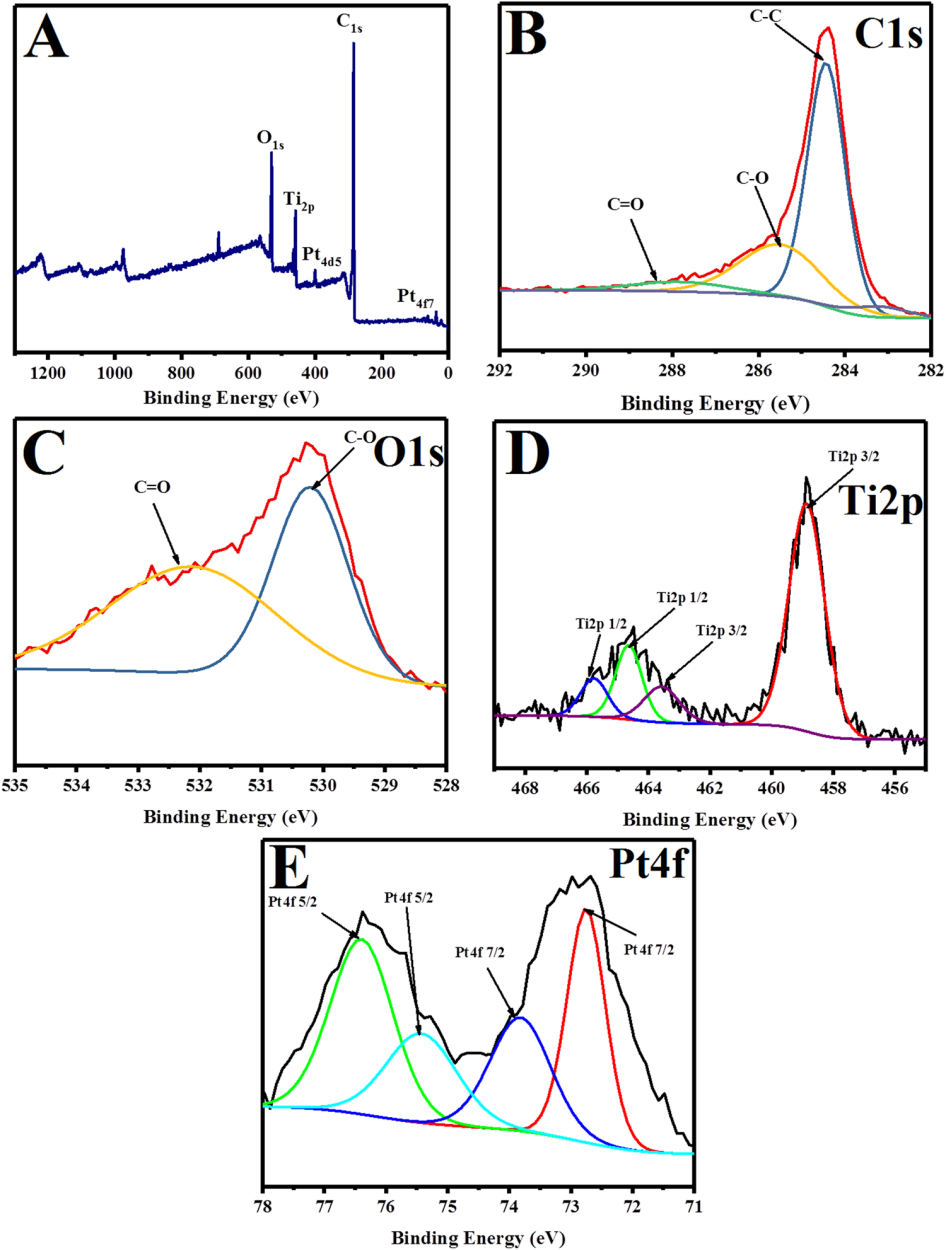
(**A**) XPS spectra survey for the synthesized hybrid graphene-Pt/TiO_2_ nanostructure (**B**) C1s spectra (**C**) O1s spectra (**D**) Ti2p spectra and (**E**) Pt4f spectra for the synthesized hybrid graphene-Pt/TiO_2_ nanostructure.

In addition, the Acknowledgements section in this Article contains errors.

“Dr. Zafar Khan Ghouri gratefully acknowledges the support of Chemical Engineering Program, Texas A&M University at Qatar and Central laboratories Unit, Qatar University.”

should read:

“Dr. Zafar Khan Ghouri and Dr. Ahmed Abdala would like to acknowledge the financial support from Texas A&M University at Qatar and Qatar Foundation.”

